# *A Fresh Start to a Healthier You!* program improves fruit and vegetable consumption and risk of food insecurity: Findings from Texas

**DOI:** 10.1016/j.pmedr.2025.103158

**Published:** 2025-06-29

**Authors:** Katelin M. Alfaro Hudak, Lindsey Breunig-Rodriguez, Renda J. Nelson, Elizabeth F. Racine

**Affiliations:** a Texas A&M AgriLife Research Center at El Paso, Texas A&M University, 1380 A&M Circle, El Paso, TX 79927, USA; b Better Living for Texans, Texas A&M AgriLife Extension Service, 2251 TAMU, College Station, TX 77843, USA

**Keywords:** Nutrition education, Better living for Texans, Supplemental nutrition assistance program-education, SNAP-Ed, Program evaluation, Outcome evaluation

## Abstract

**Objective:**

The goal of Supplemental Nutrition Assistance Program Education (SNAP-Ed) is to increase the likelihood that individuals with lower incomes who are eligible for SNAP benefits will choose healthy foods within a limited budget and be physically active. This study aimed to evaluate how participation in a Texas SNAP-Ed program *A Fresh Start to a Healthier You!* (*Fresh Start*) was associated with participants' fruit and vegetable consumption, use of food resource management strategies, and risk of food insecurity.

**Methods:**

The study used survey data collected February 2021–September 2023 from counties across Texas. This single group pre/post evaluation used generalized linear models to assess changes in outcomes from baseline to program completion. Models adjusted for age, sex, race/ethnicity, and educational attainment.

**Results:**

Participants who completed *Fresh Start* were 18–20 percentage points more likely to utilize food resource management strategies (average marginal effect [AME]: 18.2–20.0, *p* < 0.0001). The average change in being at risk of food insecurity decreased 3 percentage points (AME: 3.2, *p* = 0.012) following completion of *Fresh Start.* Measures of F&V intake significantly increased (AME: 6.7–36.3, *p* < 0.001), and the average number of days participants engaged in physical activity each week increased by 0.7 (AME: 0.7, *p* < 0.0001). Participation in *Fresh Start* was associated with improved food security, greater utilization of food resource management strategies, and increased fruit and vegetable consumption and physical activity.

**Conclusion:**

This study supports the use of SNAP-Ed and other nutrition education programs as one avenue to improve food security and diet behavior in households with lower incomes.

## Highlights

• Use of food resource management strategies increased after completing *Fresh Start*.
• Food security and diet behavior improved after *Fresh Start* program completion.
• Frequency of physical activity increased following program completion.

## 1 Introduction

The connection between poor diet quality and chronic diseases such as diabetes and cardiovascular disease is well documented (Fanelli et al., 2020). Furthermore, food insecurity (i.e., lacking consistent access to enough food for an active, healthy life (Rabbitt et al., 2024)) is associated with lower diet quality, diabetes, hypertension, hyperlipidemia, and worse overall health (Gundersen and Ziliak, 2015). Approximately 13.5 % of households in the United States (U.S.) were food insecure in 2023 (Rabbitt et al., 2024). The prevalence of food insecurity in Texas was 16.9 % from 2021 to 2023, significantly higher than the national average (Rabbitt et al., 2024).

One primary policy mechanism designed to improve food insecurity in the U.S. is the Supplemental Nutrition Assistance Program (SNAP). SNAP is the largest federal nutrition assistance program and provides funding to assist low-income households with grocery expenditures. In fiscal year (FY) 2022, approximately 88 % of eligible individuals participated in SNAP nationally. Participation rates in Texas are significantly lower than the national average, with an estimated 74 % of eligible individuals participating in SNAP in FY 2022 (Cunnyngham, 2025). Even so, Texas has the second largest SNAP-participating population; over 3 million individuals participated in FY 2024 (U.S. Department of Agriculture, Food and Nutrition Service, 2025). The Supplemental Nutrition Assistance Program Education (SNAP-Ed) complements SNAP's impact via nutrition education and obesity prevention activities (US Department of Agriculture, Food and Nutrition Service. FY, 2025). SNAP-Ed aims to improve the odds that SNAP-Ed-eligible individuals (i.e., those with low income or who live in communities where at least half of the population has low income) will “make healthy food choices within a limited budget and choose physically active lifestyles.” (US Department of Agriculture, Food and Nutrition Service. FY, 2025)

One key component of SNAP-Ed's approach is direct nutrition education classes with SNAP-Ed-eligible individuals. While the U.S. Department of Agriculture (USDA) Food and Nutrition Service establishes SNAP-Ed policy, SNAP-Ed implementation occurs at the state level via implementing agencies that utilize a variety of education programs. The local nature of SNAP-Ed is a strength as it allows for tailored activities that reflect community preferences. Yet it also necessitates sharing programs and strategies so that implementing agencies across the U.S. may learn which are effective.

Existing research on SNAP-Ed direct education suggests that it can improve food security, (Eicher-Miller et al., 2009; Rivera et al., 2016) food resource management skills (Adedokun et al., 2018; Kaiser et al., 2015), food safety practices (Adedokun et al., 2018), and fruit and vegetable intake (Dannefer et al., 2015; Hersey et al., 2015; Long et al., 2024). However, there is a paucity of peer-reviewed research evaluating SNAP-Ed direct education among adults in Texas. Because of the autonomy that implementing agencies within and between states have to develop and deliver SNAP-Ed curriculum, there is notable heterogeneity in SNAP-Ed programs across the U.S. Therefore, understanding whether improvements in diet behavior among adults are seen following participation in a Texas SNAP-Ed program is necessary. Understanding the extent to which SNAP-Ed is achieving its goals is especially important because of the high prevalence of food insecurity in Texas. Furthermore, few studies examine the connection between participation in SNAP-Ed and physical activity outcomes, and none in adults.

The Better Living for Texans (BLT) program is the SNAP-Ed program within the Texas A&M AgriLife Extension Service and Texas A&M University Department of Nutrition. BLT direct nutrition education is provided statewide by extension agents, educators, and volunteers. *A Fresh Start to a Healthier You*! (hereafter, *Fresh Start*) is the BLT program's flagship diet behavior curriculum. The goals of the program are to encourage participants to 1) increase their fruit and vegetable intake, 2) regularly engage in physical activity, 3) adopt food safety principles, and 4) use food resource management strategies to stretch their grocery budget.

No prior research has rigorously evaluated the extent to which *Fresh Start* has achieved its goals. Findings will help direct guidance and best practices for direct nutrition education within SNAP-Ed. The aim of this study is to evaluate pre/post differences in food security and diet and health behaviors among Texans who completed *Fresh Start* in 2021–2023. The hypotheses are that completion of *Fresh Start* will be associated with improved food security, greater intake of fruits and vegetables, more frequent use of food resource management behaviors, and a higher frequency of physical activity.

## 2 Methods

### 2.1 Study design

The study utilized a one-group pre/post design and compared participant outcomes at baseline with outcomes at program completion. As part of standard program operations, *Fresh Start* instructors routinely collect data from each participant, with the pre-survey administered at the beginning of the first session and the post-survey at the end of the last session. Responses to these surveys were used for analysis.

### 2.2 Study population

The program audience is SNAP-Ed-eligible adults ages 18 and over. The present study used administrative data collected as part of standard BLT procedures and includes adults (ages ≥18 years) who participated in *Fresh Start* between February 1, 2021 and September 30, 2023. Instructors delivered *Fresh Start* in 90 counties (35 %) across Texas. The study used data from all adults who completed *Fresh Start* (defined as participating in all four sessions), completed both the pre- and post-surveys, and who had non-missing age data. (to exclude any children who may have accompanied their parent/caregiver to the class and taken the survey). Of the participants who began the program and filled out the initial survey (*n* = 6313), 2170 did not attend all four sessions. Of those who completed the program, 85 had missing age data or were under age 18, resulting in 4058 individuals in the analytical sample.

### 2.3 SNAP-Ed intervention

*Fresh Start* is a four-session curriculum for adults, with sessions lasting 45–60 min. Sessions typically occur weekly, although the frequency may change to biweekly or monthly, depending on local needs and preferences. Between February 2021–September 2023, 80 % of series were taught weekly, 10 % monthly, 8 % biweekly, and the remainder (2 %) fell into the category of “other.” Sessions are usually in-person (95 % in February 2021–September 2023), although virtual or hybrid classes may be offered.

Classes include a teaching component as well as cooking demonstrations or hands-on cooking, in which the participants practice a highlighted cooking skill or recipe. Participants receive printed materials (e.g., pocket guides, recipe cards) and a nutrition education reinforcement item (e.g., measuring cups and spoons) for each lesson to reinforce key ideas.

### 2.4 Measures

The study aimed to evaluate the extent to which *Fresh Start* was achieving its goals. Therefore, the outcome variables were measures of program goals and were based on the SNAP-Ed Evaluation Framework (Food and Nutrition Service: U.S. Department of Agriculture, 2025). *Goal 1 Increase fruit and vegetable intake* Three outcomes measured fruit and vegetable intake: whether participants reported eating 1) fruit or 2) vegetables at least once a day, and 3) whether half of their plate or more was filled with fruits and vegetables for lunch and dinner. The first two outcomes were derived from two questions that asked how many days during the past seven days respondents ate fruit (or vegetables). Response options ranged from zero to seven days. These questions were based on items from the Behavioral Risk Factor Surveillance System fruit and vegetable module, which itself is based on the validated National Cancer Institute Dietary Screener Questionnaire (Centers for Disease Control and Prevention, 2024a; National Cancer Institute: Division of Cancer Control and Population Sciences, 2025). The third outcome was based on responses to the question, “Generally speaking, how much of your lunch and dinner plates are filled with fruits and vegetables?” Response options ranged from none to 3/4 of the plate, accompanied by visual representations of a plate with a shaded portion to portray a given amount. Responses were coded to indicate whether the participant filled at least half of their plate with fruits and vegetables, based on the USDA's *MyPlate* guidelines (U.S. Department of Agriculture, U.S, 2025).

*Goal 2 Regularly engage in physical activity* Participants were asked how many days in the past week they exercised for at least 30 min. This outcome was examined as a near continuous measure. *Goal 3 Adopt food safety principles* Participants reported on a 5-point Likert scale (never, rarely, sometimes, most of the time/often, always) how often they washed fresh fruits and vegetables before eating or preparing them. This measure was dichotomized to indicate whether the participant usually or always washed fruits and vegetables, where never, rarely, and sometimes were combined into the reference category.

*Goal 4 Utilize food resource management strategies* Four outcomes captured strategies for food resource management, three of which were behaviors: whether participants usually or always 1) planned meals before grocery shopping, 2) made a grocery list before shopping, and 3) compared food prices while grocery shopping. The fourth outcome was risk of food insecurity, created from the two questions from the Hunger Vital Sign (Hager et al., 2010) food insecurity screener, which has been validated for use among adults (Gundersen et al., 2017).

The primary variable of interest was an indicator of whether the outcome was reported during the pre- or post-survey. The analysis also included participant individual and household characteristics that have been found to shape nutrition behaviors, identified a priori based on previous literature (McCullough et al., 2022; Beydoun and Wang, 2008; Bitler and Seifoddini, 2019): age (in years), sex (male/female), race/ethnicity (Non-Hispanic [NH] White, NH Black, Hispanic, NH American Indian/Alaskan Native, and NH other or multiracial), educational attainment (less than high school, high school or GED, some college, college degree or higher), whether the participant or a member of their household had participated during the past 30 days in any of the following programs: SNAP, the Special Supplemental Nutrition Program for Women, Infants, and Children, Head Start, Temporary Assistance to Needy Families, and free or reduced price school meals.

### 2.5 Statistical analysis

Summary statistics of the demographic and household characteristics of all those who participated in *Fresh Start* were calculated. Significant differences between those who completed the program and those who did not were assessed using tests of proportions (categorical variables) and Wald (continuous variables) test statistics. The main analysis was restricted to participants who completed the program, had non-missing age data, and who had complete data on the outcome of interest in the pre- and post-surveys. Generalized linear models with standard errors clustered on the participant identifier and on the county where the series was taught were utilized to assess changes in outcomes from baseline to program completion. Average marginal effects are reported. Unadjusted analyses were conducted first, and then adjusted models controlled for age, sex, race/ethnicity, and educational attainment. In line with Rothman (Rothman, 1990), Perneger, (Perneger, 1998) and Rubin (Rubin, 2021), findings are reported without adjusting for multiple comparisons. An *α* of 0.05 was used and all analyses were conducted using Stata 18 (StataCorp, 2024).

## 3 Results

The majority of *Fresh Start* participants were female (75.0 %) and Hispanic (54.6 %) or non-Hispanic White (32.5 %)(Table 1). Age ranged from 18 to 96 years, and mean age was 51 years. Almost half (46.8 %) had a high school degree as their highest level of education, 20 % had some college, and 17 % had a college degree. Approximately 57 % reported receiving SNAP benefits, 26 % had children who received free or reduced price school meals, and over half (56.8 %) had utilized a food pantry or other emergency food program in the past 30 days.

**Table 1:** Baseline descriptive statistics of adult participants in Texas who completed *A Fresh Start to a Healthier You!* (*n* = 4058, 2021–2023).

	Column %^a^	SE
Age (mean, years)	51.0	0.3
Sex (male)	25.0	0.7

Race/ethnicity^b^		
White	32.5	0.8
Black	10.3	0.5
Hispanic	54.6	0.8
American Indian/Alaskan Native	0.8	0.1
Other/Multiracial	1.9	0.2

Educational attainment		
Less than high school	15.8	0.6
High school degree	46.8	0.8
Some college	20.4	0.6
College degree	17.0	0.6
Receives Supplemental Nutrition Assistance Program benefits	57.3	1.0
Children receive free/reduced price school meals	26.3	0.8
Participates in Special Supplemental Nutrition Program for Women, Infants, and Children	11.5	0.6
Participates in food pantry or other emergency food program	56.8	1.0
Children participate in Head Start	6.6	0.5
Participates in Temporary Assistance to Needy Families	1.2	0.2

a Due to rounding, column percentages may not add to 100.

b White, Black, American Indian/Alaskan Native, and Other/Multiracial include those not reporting Hispanic ethnicity. Other/Multiracial includes individuals who reported identifying as more than one race, as well as those reporting Asian or Native Hawaiian or other Pacific Islander. Because of small cell sizes, these groups were included together into one category.

At baseline, one quarter (25.7 %) of program participants reported consuming vegetables daily, and 15.6 % reported consuming fruit daily (Table 2). Less than a third (30.5 %) filled at least half of their plate with fruit and vegetables during lunch and dinner. The majority did not consistently (i.e., usually or always) utilize food resource management strategies, including plan meals (32.9 %), make a grocery list before going shopping (44.7 %), and compare grocery prices (45.7 %). Approximately 55 % were at risk of food insecurity.

**Table 2:** Descriptive statistics and associations of diet-related behavior outcomes among adult participants in Texas at baseline and following participation in *A Fresh Start to a Healthier You!* (2021–2023, n = 4058).

	Baseline		Post		Unadjusted^a^		Adjusted^b^	
	Column %	SE	Column %	SE	Average marginal effect	p-value	Average marginal effect	p-value
*Goal 1 Increase fruit and vegetable intake*								
Eats fruit daily	15.6	0.6	21.5	0.7	5.7	0.001	6.7	0.001
Eats veg daily	25.7	0.7	35.7	0.8	10.0	0.00003	10.3	0.00005
At least 1/2 plate filled with F&V	30.5	0.9	62.9	0.9	33.7	4.0e-11	36.3	1.1e-10

*Goal 2 Regularly engage in physical activity*								
Days/week exercised ≥30 min (mean)	2.0	0.0	2.6	0.0	0.7	5.9e-08	0.7	0.000003

*Goal 3 Adopt food safety principles*								
Usually or always washes F&V before eating	75.5	0.7	89.0	0.5	14.0	0.00006	13.6	0.0001

*Goal 4 Utilize food resource management strategies*								
Usually or always plans meals	32.9	0.8	50.6	0.8	17.8	2.1e-14	18.3	3.5e-14
Usually or always makes a grocery list	44.7	0.8	63.8	0.8	19.5	2.7e-10	20.0	1.7e-09
Usually or always compares grocery prices	45.7	0.8	63.2	0.8	18.1	3.9e-13	18.2	4.9e-12
At risk of food insecurity	54.6	0.8	51.7	0.8	−2.9	0.008	−3.2	0.012

F&V: Fruit and vegetables; SE: Standard error.

a Unadjusted average marginal effects and p-values were derived from generalized linear models with standard errors clustered on the county and the participant identifier. Continuous outcomes used the Gaussian family and identity link function, while categorical outcomes used the binomial family and logit link function. Analyses include participants who were ages 18 years or older and who completed the program.

b Adjusted average marginal effects and *p*-values were derived from generalized linear models with standard errors clustered on the county and the participant identifier. Analyses adjusted for age, sex, race/ethnicity, and educational attainment. Supplementary Table A1 presents the full models. Average marginal effects were calculated at the means of other model variables.

Significant improvements in each outcome were observed following completion of *Fresh Start* (Table 2). The proportion of participants who reported consuming vegetables (35.7 % at post, adjusted average marginal effect [AME]: 10.3, *p* = 0.00005) and fruit (21.5 % at post, adjusted AME: 6.7, *p* = 0.001) daily increased, and the proportion filling at least half of their plate with fruit and vegetables during lunch and dinner doubled from baseline (30.5 % to 62.9 %, adjusted AME: 36.3, *p* = 1.1e-10). Utilization of each food resource management strategy assessed increased (all *p*-values <0.0001), and the proportion of participants at risk of food insecurity decreased (54.6 % to 51.7 %, AME: −3.2, *p* = 0.012). The number of days per week that participants exercised for 30 min or more increased from 2.0 days to 2.6 days on average (adjusted AME: 0.7, *p* = 0.000003). (Table A1 in Supplemental material presents the full model results).

## 4 Discussion

In a sample of SNAP-Ed participants from Texas, completion of *Fresh Start* was associated with increased fruit and vegetable consumption, more frequent utilization of food safety and food resource management practices, increased physical activity, and a lower risk of food insecurity. These results support the study hypothesis that participation in *Fresh Start* would relate to improvements in fruit and vegetable intake, food resource management, physical activity, and food security.

No identified peer-reviewed studies have evaluated how participation in a Texas direct education SNAP-Ed program is associated with food security, nutrition behavior, or physical activity outcomes among adults. Even so, previous research has examined how participation in SNAP-Ed programs is linked to several of these outcomes in other states, described below (Rivera et al., 2019).

*Goal 1 Increase fruit and vegetable intake* Regular fruit and vegetable consumption is associated with a myriad of health benefits, including reduced risks of cardiovascular disease, stroke, and cancer (Liu, 2003; Slavin and Lloyd, 2012). As such, regular consumption of fruit and vegetables is part of the SNAP-Ed priority outcome indicators (Food and Nutrition Service: U.S. Department of Agriculture, 2025). The proportion of *Fresh Start* participants eating fruit daily, vegetables daily, and filling at least half of their plate with fruits and vegetables significantly increased from baseline to program completion.

These results align with previous research that found significantly increased fruit and vegetable consumption following participation in SNAP-Ed direct education (Dannefer et al., 2015; Hersey et al., 2015; Long et al., 2024). Results of the present study are not directly comparable to previously published literature because other studies typically assessed participants' daily intake of cups of fruit and cups of vegetables. For example, attending two or more SNAP-Ed classes at Farmers' Markets in New York City (compared with attending one or no classes) was associated with consuming 0.45 cups more of fruit and vegetables per day (Dannefer et al., 2015), and older adults participating in SNAP-Ed in Michigan increased their fruit and vegetable consumption by 0.52 cups on average (Long et al., 2024). In contrast, an evaluation of several other SNAP-Ed programs did not find significant differences in fruit and vegetable intake among young children or low-income women (Gabor et al., 2012).

*Goal 2 Regularly engage in physical activity* There is a lack of published research that links participation in a direct education SNAP-Ed program with physical activity among adults. One study examined participation in a school-based California SNAP-Ed program and physical activity among fourth and fifth grade students and did not find significant changes in physical activity (Linares et al., 2023). Another study examined the association between the presence and “dose” of physical activity-targeting SNAP-Ed programs in California and students' cardiorespiratory fitness and BMI and found that students in schools with these programs had better fitness levels on average and lower BMI z-scores (Thompson et al., 2020). The results of the present study indicate that adult participants who completed *Fresh Start* engaged in physical activity more frequently—an average of 0.6 days more per week than at baseline. The U.S. Department of Health and Human Services, 2024b recommend that adults engage in at least 150 min of moderate activity per week (U.S. Department of Health and Human Services, 2024b), and on average, *Fresh Start* participants were not meeting this recommendation, even with the increase in physical activity in the post period. Nonetheless, regularly engaging in physical activity is associated with lower risks of cardiovascular disease, type 2 diabetes, and dementia and improved mood and psychological well-being (Mikkelsen et al., 2017; Reiner et al., 2013),and recent evidence suggests that the health benefits of physical activity occur in a dose-response relationship rather than as a threshold effect (Warburton and Bredin, 2017). Therefore, an average increase of 0.6 days per week engaging in physical activity for 30 min or more can be expected to lead to an improvement in health.

*Goal 3 Adopt food safety principles* The proportion of *Fresh Start* participants who usually or always washed fruit and vegetables before eating significantly increased after completing *Fresh Start*. This finding aligns with the limited prior research assessing how participation in a SNAP-Ed program is associated with food safety practices. Adedokun and colleagues found that participation in a SNAP-Ed program in Kentucky was associated with increased utilization of two food safety practices: not leaving meat and dairy foods out for more than two hours and not thawing frozen foods at room temperature (Adedokun et al., 2018).

*Goal 4 Utilize food resource management strategies* A larger body of research has examined food resource management strategies and food security in connection with SNAP-Ed. Two previous studies found that participation in SNAP-Ed programs were associated with improvements in food resource management strategies such as planning meals ahead of time and comparing prices while grocery shopping (Adedokun et al., 2018; Kaiser et al., 2015). Both studies created summative scales of the individual strategies for the analysis; however, Kaiser and colleagues (Kaiser et al., 2015) also presented the percentage of participants reporting that they always or mostly practiced each strategy in the pre- and post-period, allowing for comparison of results from the current study. Mean differences were similar for each of the three common strategies (planning meals, making a grocery list, and comparing grocery prices). For example, the pre/post difference in the percentage of participants in FY13 reporting that they always or most of the time 1) planned meals was 19.0 and 2) shopped with a list was 18.5, compared to 17.8 and 19.5, respectively, in our study.

Improvements in food resource management strategies can help families stretch their food budgets, which in turn has the potential to alleviate food insecurity. Indeed, *Fresh Start* participants at risk of food insecurity decreased from 54.6 % at baseline to 51.7 % at program completion. Greater utilization of food resource management strategies was associated with significantly reduced risk of running out of food among California SNAP-Ed participants (Kaiser et al., 2015). Similarly, participation in Indiana SNAP-Ed improved food security both in the short term (immediately following program completion) (Eicher-Miller et al., 2009) and in the long-term, i.e., one year later (Rivera et al., 2016).

### 4.1 Strengths and limitations

The present study has several notable strengths, including having a large sample of Texans from across the state, a high proportion of completers, and a range of outcomes, including a measure of physical activity. Even so, this study has at least the following four limitations. First, although the sample was large and included individuals from across the state, it is not representative of the Texan or U.S. population. Therefore, the study findings are not necessarily generalizable across other samples. Second, the study did not have a comparison group, hindering the ability to draw conclusions regarding causality. One drawback to single group pre-post designs is the testing threat, which may have biased study results away from the null hypothesis. That is, taking the pretest at the beginning of the *Fresh Start* program may have primed participants for the program and for the post test, so that participants responded differently on the post test than they would have otherwise. In addition, several developments in SNAP occurred during the study period. Notably, the Thrifty Food Plan (which serves as the basis for SNAP benefit amounts) was reevaluated, and for the first time, did not have to meet the cost neutrality constraint. As a result, the monthly SNAP benefit amounts increased an average of $36 per person, effective October 1, 2021 (.S. Department of Agriculture, 2021a; U.S. Department of Agriculture, 2021b). In addition, the temporary increase in SNAP benefits as part of Covid-19 recovery efforts ended in March 2023, resulting in a monthly decrease in benefits of $95 per household (U.S. Department of Agriculture, 2023). These changes could have influenced the nutrition behavior outcomes assessed in this evaluation. Nonetheless, the short duration of *Fresh Start* (i.e., four weeks for most participants) limits the extent to which policy changes would occur during program participation and potentially affect outcomes. Future evaluations should include a comparison group to mitigate the testing threat, as well as changes over time that could affect participants' diet and health behaviors independent of the program.

Third, the analysis only included those who completed *Fresh Start.* Completers and non-completers differed on several observable characteristics (Table A2 in the Supplemental material). Completers were significantly more likely to be female (*p* = 0.000), have children that received free or reduced-price school meals (*p* = 0.009), and to utilize a food pantry or other emergency food program (p = 0.000). Completers were also older on average (51.0 vs. 48.1) and were less likely to receive SNAP benefits (p = 0.000). Importantly, it is possible that those who completed the program were more motivated to change their health behavior or were otherwise different in unobservable ways than those who did not complete the program. If this is the case, then attrition may be biasing study results. Fourth, participants completed the post test immediately following program completion. And although significant improvements were seen in all outcomes examined, it is unknown whether these improvements will be sustained over time. Future research should assess outcomes over the long term.

### 4.2 Conclusions

This study adds to the existing literature by evaluating how participation in a SNAP-Ed program in Texas was associated with a range of health behavior outcomes, including a measure of physical activity. Completion of the Better Living for Texans program *A Fresh Start to a Healthier You!* was associated with significant increases in participant fruit and vegetable consumption, physical activity, adoption of food safety principles, and utilization of food resource management strategies and a significant reduction in the risk of food insecurity. Nutrition education provided via SNAP-Ed can provide knowledge and tools to help SNAP-eligible individuals and families make diet and behavioral choices that support a healthy lifestyle, including using strategies to make healthy choices within a limited budget. This study supports the use of SNAP-Ed as one avenue to improve food security and diet behavior in households eligible for SNAP benefits.

## Funding disclosures

This research did not receive any specific grant from funding agencies in the public, commercial, or not-for-profit sectors.

## CRediT authorship contribution statement

**Katelin M. Alfaro Hudak:** Writing – original draft, Methodology, Formal analysis, Conceptualization. **Lindsey Breunig-Rodriguez:** Writing – original draft, Data curation. **Renda J. Nelson:** Writing – review & editing, Supervision, Data curation, Conceptualization. **Elizabeth F. Racine:** Writing – review & editing, Supervision, Conceptualization.

## Ethics statement

The Texas A&M University Institutional Review Board determined that this study met the criteria for Exemption.

## Declaration of competing interest

The authors declare the following financial interests/personal relationships which may be considered as potential competing interests: Renda J. Nelson and Lindsey Breunig-Rodriguez were employed by Texas A&M AgriLife Extension Service and received funding from the Better Living for Texans program at the time of data collection and during manuscript preparation. Katelin M. Alfaro Hudak and Elizabeth F. Racine have no conflicts of interest to report.

## Data Availability

The data that has been used is confidential.

## Appendix A Supplementary data

Supplementary material: Full regression results and comparison of program completers and non-completers. Image 1
