# Supplementary material for: A Fresh Start to a Healthier You! program improves fruit and vegetable consumption and risk of food insecurity: Findings from Texas
Source: Prev Med Rep. 2025 Jun 29;56:103158. doi: 10.1016/j.pmedr.2025.103158 (PMC12271807; doi:10.1016/j.pmedr.2025.103158)
Supplement: Supplementary file 1 — Supplementary material: Full regression results and comparison of program completers and non-completers. [file mmc1.docx]

*A Fresh Start to a Healthier You*! program improves fruit and vegetable consumption and risk of food insecurity: Findings from Texas

**Table A1.** Adjusted analyses of diet-related behavior outcomes from baseline to post participation in *A Fresh Start to a Healthier You!* ^a^

|  | (1) | (2) | (3) | (4) | (5) | (6) | (7) | (8) | (9) |
| --- | --- | --- | --- | --- | --- | --- | --- | --- | --- |
|  | Number of days/week  exercised ≥30 minutes | Eats fruit daily | Eats veg daily | At least ½ plate filled with F&V | Washes F&V | Plans meals | Makes grocery list | Compares grocery prices | At risk of food insecurity |
|  |  |  |  |  |  |  |  |  |  |
| Post program | 0.65*** | 0.47*** | 0.50*** | 1.47*** | 1.03*** | 0.75*** | 0.81*** | 0.73*** | -0.13* |
|  | (0.38 - 0.92) | (0.25 - 0.69) | (0.28 - 0.72) | (1.08 - 1.86) | (0.62 - 1.44) | (0.54 - 0.96) | (0.55 - 1.06) | (0.53 - 0.94) | (-0.23 - -0.03) |
| Age | -0.01** | 0.01* | 0.01** | 0.01* | 0.01 | -0.01 | 0.00 | 0.00 | -0.00 |
|  | (-0.02 - -0.00) | (0.00 - 0.01) | (0.00 - 0.01) | (0.00 - 0.02) | (-0.00 - 0.02) | (-0.01 - 0.00) | (-0.00 - 0.01) | (-0.00 - 0.01) | (-0.01 - 0.01) |
| Sex (male) | 0.47*** | -0.27 | -0.00 | -0.57*** | -0.83*** | -0.33*** | -0.62*** | -0.49*** | -0.07 |
|  | (0.19 - 0.74) | (-0.59 - 0.06) | (-0.20 - 0.19) | (-0.80 - -0.34) | (-1.00 - -0.67) | (-0.44 - -0.23) | (-0.80 - -0.43) | (-0.62 - -0.36) | (-0.34 - 0.20) |
| Race/ethnicity |  |  |  |  |  |  |  |  |  |
| NH White | Ref. | Ref. | Ref. | Ref. | Ref. | Ref. | Ref. | Ref. | Ref. |
| NH Black | -0.14 | -0.10 | -0.19 | -0.01 | -0.00 | -0.24 | -0.51** | -0.34 | 0.49* |
|  | (-0.64 - 0.36) | (-0.66 - 0.46) | (-0.46 - 0.09) | (-0.32 - 0.31) | (-0.53 - 0.52) | (-0.52 - 0.05) | (-0.84 - -0.19) | (-0.75 - 0.08) | (0.03 - 0.95) |
| Hispanic | -0.11 | -0.46* | -0.66*** | -0.71* | 0.33 | 0.08 | -0.11 | 0.18 | 0.23 |
|  | (-0.53 - 0.32) | (-0.85 - -0.07) | (-0.96 - -0.36) | (-1.37 - -0.06) | (-0.14 - 0.81) | (-0.38 - 0.54) | (-0.42 - 0.19) | (-0.14 - 0.51) | (-0.48 - 0.93) |
| NH AIAN | 0.46 | 0.06 | -0.02 | 0.34 | 0.56 | 0.12 | 0.23 | -0.02 | -0.00 |
|  | (-0.40 - 1.31) | (-0.84 - 0.96) | (-0.70 - 0.66) | (-0.78 - 1.45) | (-0.32 - 1.45) | (-0.39 - 0.63) | (-0.38 - 0.85) | (-0.62 - 0.58) | (-0.62 - 0.62) |
| NH Other | 1.05*** | 0.67* | 0.22 | 0.83** | 0.62* | 0.16 | 0.03 | 0.53* | -0.04 |
|  | (0.43 - 1.66) | (0.07 - 1.26) | (-0.28 - 0.72) | (0.33 - 1.34) | (0.02 - 1.21) | (-0.32 - 0.64) | (-0.45 - 0.51) | (0.10 - 0.97) | (-0.52 - 0.44) |
|  |  |  |  |  |  |  |  |  |  |
| Educational attainment |  |  |  |  |  |  |  |  |  |
| < High school | Ref. | Ref. | Ref. | Ref. | Ref. | Ref. | Ref. | Ref. | Ref. |
| High school | -0.10 | -0.42** | -0.27* | -0.19 | 0.23 | 0.23 | 0.27* | 0.08 | -0.43** |
|  | (-0.41 - 0.22) | (-0.71 - -0.12) | (-0.48 - -0.06) | (-0.65 - 0.27) | (-0.04 - 0.49) | (-0.04 - 0.50) | (0.04 - 0.50) | (-0.17 - 0.33) | (-0.74 - -0.12) |
| Some college | 0.15 | -0.18 | -0.09 | -0.04 | 0.47** | 0.38*** | 0.56*** | 0.33** | -0.54*** |
|  | (-0.15 - 0.45) | (-0.49 - 0.12) | (-0.30 - 0.12) | (-0.39 - 0.32) | (0.14 - 0.80) | (0.18 - 0.58) | (0.34 - 0.77) | (0.11 - 0.55) | (-0.78 - -0.29) |
| ≥ College degree | 0.34* | -0.12 | -0.13 | 0.36** | 0.52** | 0.40*** | 0.73*** | 0.24* | -1.12*** |
|  | (0.03 - 0.65) | (-0.41 - 0.17) | (-0.35 - 0.09) | (0.09 - 0.64) | (0.18 - 0.86) | (0.17 - 0.63) | (0.55 - 0.90) | (0.00 - 0.48) | (-1.41 - -0.84) |
| Constant | 2.52*** | -1.56*** | -0.92*** | -0.87** | 0.52* | -0.62** | -0.42* | -0.46* | 0.54* |
|  | (1.76 - 3.28) | (-2.09 - -1.03) | (-1.27 - -0.57) | (-1.46 - -0.28) | (0.12 - 0.92) | (-1.05 - -0.20) | (-0.80 - -0.03) | (-0.83 - -0.08) | (0.00 - 1.09) |
|  |  |  |  |  |  |  |  |  |  |

Robust 95% CI in parentheses

*** p<0.001, ** p<0.01, * p<0.05

^a^Results are from adjusted generalized linear models with standard errors clustered on the county and the participant identifier. Continuous outcomes used the linear regression function (Gaussian family and identity link function), while categorical outcomes used the binomial family and logit link function. Analyses include participants who were ages 18 years or older and who completed the program.

AIAN: American Indian/Alaskan Native; CI: confidence interval; F&V: fruits and vegetables; NH: Non-Hispanic; Ref.: reference category

**Table A2 .** Comparison of baseline characteristics of adult participants in Texas who completed *A Fresh Start to a Healthier You!* and those who did not complete the program (2021-2023)

|  | Total | | Completer^a^ | | Non-completer^a^ | | |  | |
| --- | --- | --- | --- | --- | --- | --- | --- | --- | --- |
|  | n=6,207 | | n=4,058 | | n=2,149 | | |  | |
|  | Column %^b^ | SE | Column %^b^ | SE | Column %^b^ | SE | p-value^c^ | |  |
| Age (mean, years) | 50.0 | 0.2 | 51.0 | 0.3 | 48.1 | 0.4 | 0.000 | |  |
| Sex (male) | 28.7 | 0.6 | 25.0 | 0.7 | 35.7 | 1.0 | 0.000 | |  |
| Race/ethnicity^d^ |  |  |  |  |  |  |  | |  |
| White | 33.0 | 0.6 | 32.5 | 0.8 | 34.0 | 1.1 | 0.243 | |  |
| Black | 9.8 | 0.4 | 10.3 | 0.5 | 8.8 | 0.6 | 0.077 | |  |
| Hispanic | 54.6 | 0.7 | 54.6 | 0.8 | 54.4 | 1.1 | 0.869 | |  |
| American Indian/Alaskan Native | 0.7 | 0.1 | 0.8 | 0.1 | 0.6 | 0.2 | 0.388 | |  |
| Other/Multiracial | 2.0 | 0.2 | 1.9 | 0.2 | 2.2 | 0.3 | 0.352 | |  |
| Educational attainment |  |  |  |  |  |  |  | |  |
| Less than high school | 16.0 | 0.5 | 15.8 | 0.6 | 16.4 | 0.8 | 0.603 | |  |
| High school degree | 45.1 | 0.6 | 46.8 | 0.8 | 41.8 | 1.1 | 0.000 | |  |
| Some college | 21.5 | 0.5 | 20.4 | 0.6 | 23.7 | 0.9 | 0.003 | |  |
| College degree | 17.4 | 0.5 | 17.0 | 0.6 | 18.1 | 0.9 | 0.294 | |  |
| Receives Supplemental Nutrition Assistance Program benefits | 60.8 | 0.8 | 57.3 | 1.0 | 68.5 | 1.3 | 0.000 | |  |
| Children receive free/reduced price school meals | 25.1 | 0.7 | 26.3 | 0.8 | 22.4 | 1.2 | 0.009 | |  |
| Participates in Special Supplemental Nutrition Program for Women, Infants, and Children | 12.0 | 0.5 | 11.5 | 0.6 | 13.2 | 1.0 | 0.118 | |  |
| Participates in food pantry or other emergency food program | 50.9 | 0.8 | 56.8 | 1.0 | 38.0 | 1.4 | 0.000 | |  |
| Children participate in Head Start | 6.7 | 0.4 | 6.6 | 0.5 | 7.0 | 0.7 | 0.654 | |  |
| Participates in Temporary Assistance to Needy Families | 1.3 | 0.2 | 1.2 | 0.2 | 1.4 | 0.3 | 0.699 | |  |

^a^Completers participated in all four *Fresh Start* sessions. Non-completers began the program but did not finish.

^b^Due to rounding, column percentages may not add to 100.

^c^Tests of significance assessed differences in characteristics between completers and non-completers. Tests of proportions were used for all variables except age, for which a Wald test was used.

^d^White, Black, American Indian/Alaskan Native, and Other/Multiracial include those not reporting Hispanic ethnicity. Other/Multiracial includes individuals who reported identifying as more than one race, as well as those reporting Asian or Native Hawaiian or other Pacific Islander. Because of small cell sizes, these groups were included together into one category.
